# The art of taking sides: Key determinants for the polar localization of silicon transporter

**DOI:** 10.1093/plcell/koad079

**Published:** 2023-03-17

**Authors:** Ching Chan

**Affiliations:** The Plant Cell, American Society of Plant Biologists, USA; Department of Life Science, National Taiwan Normal University, Taipei 11677, Taiwan

Membrane-localized transporters are critical for plant uptake of mineral nutrients. Instead of being evenly distributed on the membranes, some transporter proteins display asymmetric organization/polar localization in specific tissues, which facilitates directional transport toward the central vasculature (Naramoto 2017). The molecular mechanisms for the establishment, maintenance, and dynamic modulation of transporter polar localization, however, remain largely unexplored. The silicon uptake channel Lsi1 (Ma et al. 2006) and the exporter Lsi2 (Ma et al. 2007) are two examples of polarly localized transporters. Using quantitative image analyses with a comprehensive set of deletion and mutagenesis constructs, **Noriyuki Konishi and colleagues** (Konishi et al. 2023) reported essential residues for the asymmetric distribution of OsLsi1 in this issue of *The Plant Cell*.

*OsLsi1* encodes an aquaporin that is localized at the distal side of root exodermis and endodermis (Ma et al. 2006) (see Figure). Deletion of the *N*-terminus alone did not affect the localization pattern of OsLsi1. On the other hand, while deletion of the *C*-terminus resulted in partial accumulation of the protein in the endoplasmic reticulum (ER), the polar localization of plasma membrane-associated OsLsi1 was maintained. Interestingly, when both the *N*- and *C*-terminal regions were deleted, polar localization of OsLsi1 was abolished in both the exodermis and endodermis (Figure). Therefore, both the *N*- and *C*-terminal contain signals that determine OsLsi1 asymmetric distribution. In addition, the *C*-terminus was required for trafficking of OsLsi1 from the ER to the plasma membrane. This notion was further confirmed by the creation of a protein chimera between OsLsi1 and OsNIP1;1. OsNIP1;1 does not show polar localization in its native form. But when fused with the *C*-terminus of OsLsi1, the chimeric protein was observed at the distal side of the exodermis and endodermis, indicating that the *C*-terminal region of OsLsi1 is sufficient to mediate polar localization.

**Figure.**
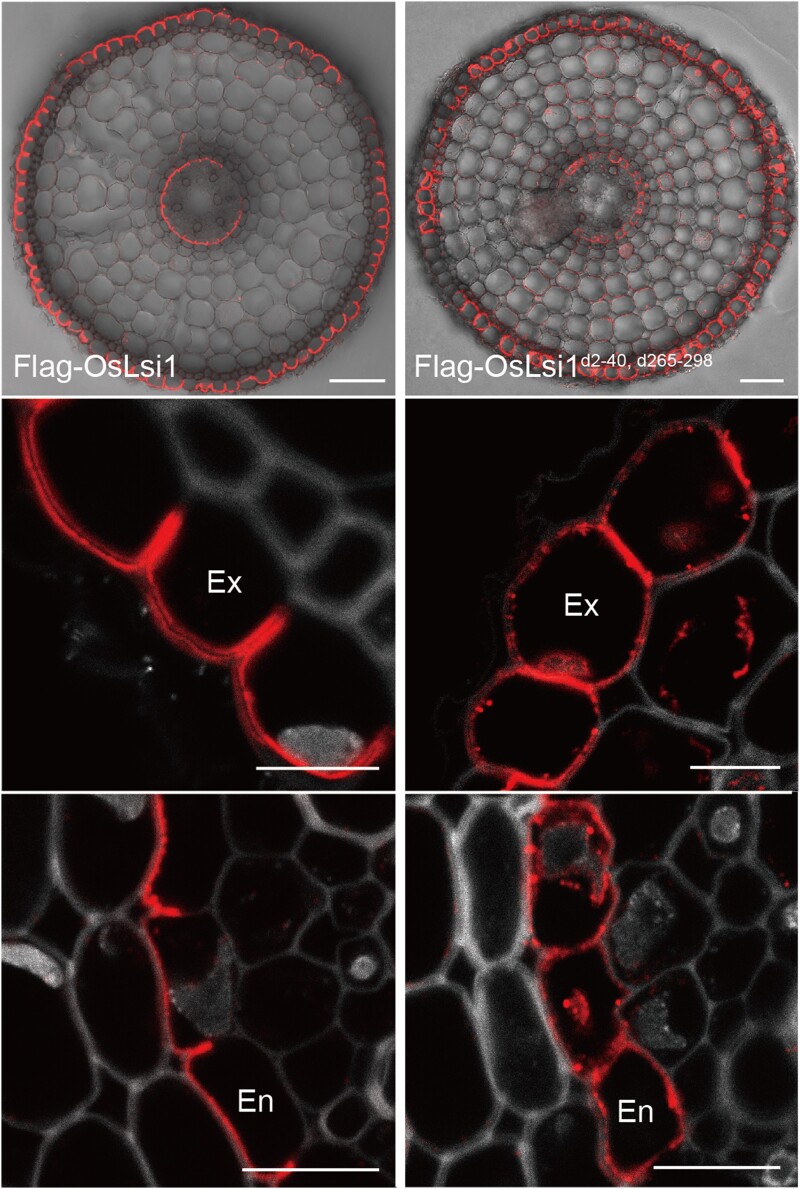
The *N*- and *C*-terminal regions determine OsLsi1 polar localization. OsLsi1 localized on the distal side of the rice root exodermis and endodermis (left). Deletion of both the *N*- and *C*-terminal regions abolished OsLis1 polar localization (right). The lower panels show enlargements of exodermis (Ex) and endodermis (En) from the respective top panels. Reprinted from Konishi et al. (2023)Fig. 1B and E.

The authors performed site-directed mutagenesis experiments to narrow down the region determining OsLsi1 asymmetric distribution. Overall, two isoleucine residues (Ile18 at the *N*-terminus and Ile285 at the *C*-terminus) and a positively charged cluster (arginine and lysine residues at the *C*-terminus) were shown to be critical for OsLsi1 asymmetric distribution. In contrast, mutations in potential sites of phosphorylation (tyrosine and serine residues) and lysine modification did not affect OsLsi1 polar localization, indicating that post-translational modification is not important in this process. Finally, the physiological function of these amino acids was confirmed in planta. When both Ile18 and Ile285 were mutated, OsLsi1 polar localization was compromised and silicon uptake was significantly reduced, highlighting the importance of the polar localization of OsLsi1 in efficient silicon uptake.

Although silicon is not considered an essential element for plant growth, external silicon supply promotes plant performance under various abiotic and biotic stresses (Ma and Yamaji 2015). Notably, silicon application promotes broad-spectrum disease resistance without growth-defense trade-offs (Van Bockhaven et al. 2013). The regulation of silicon uptake in planta is thus important in practical application. It will be interesting to further explore how specific amino acids contribute to the asymmetric organization and the tissue-specific function of these transporters at the exodermis and endodermis.
